# Supplementary material for: Hand Hygiene, Face Mask Use, and Associated Factors during the COVID-19 Pandemic among the Students of Mongar Higher Secondary School, Bhutan: A Cross-Sectional Study
Source: Int J Environ Res Public Health. 2023 Jan 6;20(2):1058. doi: 10.3390/ijerph20021058 (PMC9859439; doi:10.3390/ijerph20021058)
Supplement: Supplementary file 1 [file ijerph-20-01058-s001.zip › ijerph-2068678-supplementary.pdf]

Supplementary Tables

Table S1. Collinearity of factors included in the good hand-hygiene model using variance inflation factor.

Variable	VIF	1/VIF
Sex		
Male	1.03	0.943743
Grade		
10	1.75	0.571591
11	3.02	0.331462
12	2.80	0.356647
Father education		
Primary	1.18	0.847548
High school	1.63	0.615099
Diploma	1.40	0.713282
Bachelor and above	1.44	0.694565
Father occupation		
Civil Servant	2.08	0.481363
Private employee	1.16	0.863512
Driver	1.03	0.973201
Others	1.09	0.915655
Mean VIF	1.63	

Table S2. Collinearity of factors included in the good face mask use model using variance inflation factor.

Variable	VIF	1/VIF
Sex		
Male	1.01	0.992360
Boarder		
Yes	1.33	0.753331
Father occupation		
Civil Servant	1.36	0.735063
Private employee	1.15	0.869105
Driver	1.02	0.97022
Others	1.11	0.903781
Mean VIF	1.16	

Table S3. Good hand hygiene stratified by grades among students of Mongar Higher Secondary School, Bhutan.

Grades	Good n(%)	Bad n(%)
9	24 (45.3)	29 (54.7)
10	11 (24.4)	34 (75.6)
11	90 (31.8)	193 (68.2)

12	53 (35.3)	97 (64.7)

Table S4. Good mask use stratified by grades among students of Mongar Higher Secondary School, Bhutan.

Grades	Good mask use n (%)	Bad n(%)
9	12 (22.6)	41 (77.4)
10	8 (17.8)	37 (82.2)
11	71 (25.1)	212 (74.9)
12	27 (18.0)	127 (18.0)
